# Celiac Disease and Cardiovascular Risk: A Retrospective Case-Control Study

**DOI:** 10.3390/jcm12062087

**Published:** 2023-03-07

**Authors:** Maria Pina Dore, Sandro Mereu, Pier Sergio Saba, Michele Portoghese, Giovanni Mario Pes

**Affiliations:** 1 Dipartimento di Medicina, Chirurgia e Farmacia, University of Sassari, Viale San Pietro 43, 07100 Sassari, Italy; 2 Baylor College of Medicine, One Baylor Plaza, Houston, TX 77023, USA; 3 Clinical and Interventional Cardiology, Sassari University Hospital, 07100 Sassari, Italy; 4 Cardiovascular Surgery Unit, Sassari University Hospital, 07100 Sassari, Italy; 5 Sardinia Longevity Blue Zone Observatory, 08040 Ogliastra, Italy

**Keywords:** celiac disease, cardiovascular disease, intima-media thickness, atherosclerosis, gluten-free diet

## Abstract

Background: The association of celiac disease (CD) with premature atherosclerosis, including increased carotid artery intima-media thickness and cardiovascular disease (CVD), is controversial. The aim of this study was to investigate this relationship. Methods: Clinical records of patients from Northern Sardinia referred to the Gastroenterology section of the Department of Medicine, University of Sassari, Italy, were analyzed. Unadjusted and adjusted odds ratios (ORs) for CVD with their 95% confidence intervals (CIs) were calculated according to established risk factors, including age, sex, diabetes, dyslipidemia, overweight/obesity, blood hypertension, and cigarette smoking, as well as a possible risk factor such as H. pylori infection. Results: In a total of 8495 patients (mean age 52.1 ± 17.3 years; 64.7% females), 2504 reported a diagnosis of CVD and 632 of CD. Logistic regression analysis showed a significantly reduced risk of CVD among patients with CD (OR 0.30, 95% CI 0.22–0.41). Moreover, the long duration of the gluten-free diet (GFD) was able to lower the risk of CVD in celiac patients. Finally, CD significantly decreased the frequency of carotid plaques (11.8% vs. 40.1%, *p* < 0.001). Conclusions. Our retrospective study demonstrated that CD reduces the risk of CVD in general and more specifically of carotid lesions after adjusting for potential confounders, especially in those on GFD for a long time.

## 1. Introduction

Celiac disease (CD) is defined as a chronic immune-mediated inflammation causing injury in the small intestine, resulting from exposure to gluten in genetically predisposed individuals [1]. Although historically the disease has been described as a primary malabsorption syndrome characterized by diarrhea, steatorrhea, weight loss, nutritional deficiencies, and osteomalacia, in recent decades the clinical presentation has appeared extremely heterogeneous, making the conventional definition rather obsolete [2,3]. Actually, there is a current tendency to interpret the disease as a broader condition in which the small intestine is only one of the organs affected [4,5]. This reappraisal of CD as a systemic disorder has resulted in an emerging interest in the extraintestinal manifestations of the disease [6]. This prompted researchers to pose the question of whether, in the long run, the CD could actually constitute a risk factor for disorders involving other organs besides the gut, such as the nervous system [7], the endocrine system [8], the cardiovascular system [9,10], the reproductive system [11], the skin [12] and the immune system [13,14].

The possible association between CD and cardiovascular disease (CVD) has aroused conspicuous interest since CVD is globally the first cause of death, as well as the most avoidable risk by means of a scrupulous preventive strategy [15]. Following the first study by West et al. in 2004 [16], which aimed to quantify the risk of malignancy and mortality in CD patients, a number of case-control studies [17,18,19] prospective studies [20,21,22] and meta-analyses [23,24,25] have been conducted to ascertain if CD patients are at risk of developing CVD. However, despite the steady growth of the literature on this topic, opinions regarding the occurrence of CVD in patients with childhood or adult-onset CD are still conflicting, and, currently, there is no consensus among gastroenterologists and cardiologists about the risk extent [26,27,28]. An additional difficulty lies in the fact that the cohorts of CD patients include both newly diagnosed cases and those on the gluten-free diet (GFD), whose duration may be variable from a few months to many years. Moreover, CD presenting atypically is often underdiagnosed [29,30].

The global prevalence of CD in the western world was estimated at 1.4% based on serological data and 0.7% based on biopsies [31], and as high as 2.4% in Finland [32], with it being lower in southern Europe. In Italy at the end of 2019, there were 233,147 diagnosed celiacs, of which 34% belonged to the male population (78,248) and 66% to the female population (154,899). Among the Italian regions with the highest prevalence, the island of Sardinia displays a frequency of 1.43% [33], being the fourth among all the Italian regions. Since the prevalence of CD varies according to latitude, there should also be a large amount of ethnic and genetic heterogeneity among the different cohorts investigated, which may introduce a confounding element distorting the correct interpretation of study findings.

Sardinia is an Italian island characterized by a homogenous genetic background, including a very high frequency of class II HLA–DQ haplotypes that predispose to CD, second only to the African population of Saharawi [34]. Because of that, the Sardinian population and physicians are very sensitive to CD screening, even in general medicine, making the underdiagnosis of CD a rare event.

Taking advantage of this special setting, this study aimed to investigate the association between CD and CVD risk, adjusting for several potential confounders and the duration of GFD in a large cohort of patients.

## 2. Materials and Methods

### 2.1. Study Design

This was a retrospective, single-center, case-control study. Patients with CVD were considered cases, and patients without CVD were considered controls. Among the major risk factors for CVD, the CD was also included. Patients undergoing an upper endoscopy were recruited in a tertiary referral Gastroenterology Section (University of Sassari, Italy).

### 2.2. Data Collection

Data were collected from the clinical records of patients who complained of gastrointestinal symptoms between 2002 and 2019, whenever a comprehensive and accurate medical history was available. Information was gathered about sex, age, anthropometric parameters, and established CV risk factors. Furthermore, given that a long-lasting H. pylori infection was reported to be associated with an increased risk for CVD [35,36], this covariate was included in the analysis. Part of the database has previously been used for studies involving CD [37]. The CVD was stratified according to the main diagnosis into (i) atherosclerosis, (ii) arrhythmias, (iii) valvular heart disease, (iv) cardiomyopathies, and (v) hypertension (including hypertrophic cardiomyopathy).

More specifically, hypertension was defined as systolic blood pressure ≥140 mm Hg and/or diastolic blood pressure ≥90 mm Hg in at least three measurements and matched with antihypertensive treatment [38].

The presence of type 1 and type 2 diabetes was considered a single variable. Dyslipidemia was defined when plasma low-density lipoprotein cholesterol levels were higher than the desired value of 100 mg/dL, the high-density lipoprotein cholesterol was lower than 40 mg/dL, and/or triglycerides were higher than 150 mg/dL, as recommended by the ACC/AHA guidelines [39].

Diagnoses of CV disorders, high blood pressure, hypercholesterolemia, and diabetes were made by the specific specialist according to guidelines progressively developed and followed in clinical practice.

According to their smoking habits, patients were stratified as never, former, or current smokers. The body mass index (BMI) was calculated using the formula weight (kg)/height (m)², and obesity was defined as a BMI ≥30 kg/m^2^.

Since a statistically significant association between a long-lasting H. pylori infection (detection of atrophy, metaplasia, and/or dysplasia in the gastric specimens) and the presence of blood hypertension and carotid plaques was previously observed [35], this variable was included in the analysis. The duration of GFD was expressed in months.

A focal atheromatous structure encroaching the arterial lumen by at least 0.5 mm or 50% of the surrounding intima-media thickness (IMT) value or thickness equal to or exceeding 1.5 mm was defined as carotid plaques.

### 2.3. Celiac Disease

The diagnosis of CD was made according to the international guidelines progressively developed. Briefly, patients with gastrointestinal and/or extraintestinal symptoms suggestive of CD and/or manifestations of primary malabsorption were assessed for serology markers, including the total level of IgA [40]. Moreover, in patients with dyspeptic symptoms (abdominal pain or discomfort and/or diarrhea) undergoing an upper endoscopy, biopsy specimens from the duodenal mucosa, even when macroscopically normal, were collected for morphology examination [41]. An additional criterion to rule out or confirm CD was the presence of other autoimmune disorders or a positive family history for CD. Information about gluten-containing or gluten-free diets was also retrieved from the clinical records. All tests were performed on patients exposed to a gluten-containing diet for at least 4 weeks. 

### 2.4. Carotid Ultrasonography

The procedure was performed by a trained sonographer in a subgroup of 333 patients using linear-array transducers with a frequency of emission of 7.5 MHz, as previously described [35]. The IMT and the diameters were measured at the common carotid artery, bifurcation, on both sides, and internal and external carotid arteries [42].

### 2.5. Statistical Analysis

Differences between cases and controls were evaluated by the Student’s *t*-test for continuous variables and by the Pearson chi-square test for categorical variables. The association between CD and CVD was analyzed using univariable and multivariable logistic regression models. Unadjusted and adjusted odds ratios (ORs) and their 95% confidence intervals (CI) were calculated. The Hosmer–Lemeshow goodness-of-fit test was used to evaluate the fit of the models. All statistical analyses were performed using SPSS statistical software (version 22.0, Chicago, IL, USA). Additionally, *p*-values lower than 0.05 were considered statistically significant.

### 2.6. Ethical Considerations

The study was approved by the local ethics committee of “*Azienda Ospedaliero Universitaria di Sassari*” (Prot N° 2113/CE, 2014).

## 3. Results

A total of 8495 clinical records were retrieved for the analysis (mean age: 52.1 ± 17.3 years; 64.7% females). Among them, 632 patients were positive for CD, and 2503 had a specific diagnosis of CVD (29.5%). Table 1 shows the features of the study participants.

As expected, males were more numerous among cases (patients with CVD) (*p* < 0.0001). Moreover, CVD patients were nearly 20 years older (65.4 ± 11.8 vs. 46.5 ± 16.1 y, *p* < 0.0001) and more overweight/obese (BMI ≥ 25 kg/m^2^) (17.0% vs. 9.4%, *p* < 0.0001) compared with controls. Similarly, current and former smokers were more represented among cases than among controls. Interestingly, the frequency of CD patients was only 2.2% among those with a diagnosis of CVD, while it was 9.5% among controls, i.e., a value consistent with previous results observed in the same population [8]. The prevalence of dyslipidemia was threefold higher in patients with CVD compared with patients without CVD (20.1% vs. 6.8%) (Table 1). Similarly, the frequencies of diabetes and a long-lasting H. pylori infection appeared to both increase in CVD patients.

According to the distribution of the different CV disorders in the 55 cases of CD listed in Table 2, atherosclerotic lesions appeared less represented in CD patients than in subjects without CD (*p* < 0.0001).

Celiac patients were further stratified based on GFD duration (Table 3). Patients with a new CD diagnosis and on a free diet, as well as CD patients on a GFD for less than 12 months, were more prone to have CVD.

The prevalence of risk factors for CVD in celiac and non-celiac patients is shown in Table 4. All conventional risk factors, including overweight, obesity, cigarette smoke, and dyslipidemia, were statistically more prevalent in patients without CD, whereas diabetes was more common among celiac patients. In fact, type 1 diabetes is the autoimmune disorder most frequently occurring in CD patients. [14]. Moreover, a possible risk factor for CVD, such as the long-lasting H. pylori infection, was less common in subjects with CD (16% vs. 24.7%, *p* < 0.001).

The results of univariable and multivariable logistic regression analysis are shown in Table 5. In the model, the presence of CVD was used as the outcome and CD as exposure.

As expected, male sex, age, overweight/obesity, cigarette smoking, dyslipidemia, and diabetes were strong risk factors, whereas a long-lasting H. pylori infection was a mild risk factor. Instead, the presence of CD significantly reduced the risk of CVD both in the univariable analysis (OR 0.21, 95% CI 0.16–0.28) and after adjusting for the other covariates (OR 0.30, 95% CI 0.22–0.41).

The analysis conducted separately in the two sexes confirmed the inverse association between CD and the total CVD risk (OR 0.35 and 0.18 in males and females, respectively).

Ultrasonographic carotid parameters were available in a subgroup of 333 patients from the total studied cohort and are reported in Table 6 according to the presence or absence of CD. The overall prevalence of CD was 10.2%. A statistically significantly lower prevalence of right, left, and any carotid plaque as well as a lower IMT were found in CD patients.

## 4. Discussion

It is widely known that CD can be associated with multiple organ involvement, which justifies its classification as a systemic disorder [5]. Aside from its association with other autoimmune diseases such as type 1 diabetes, Hashimoto’s thyroiditis, and primary sclerosing cholangitis [8,14,43], usually explained by the sharing of various immune mechanisms [44], the potential association between CD and CVD has gained a lot of interest during the past decade, and the literature is currently recording an exponential growth of publications on this specific field. Most studies in this area, whether observational studies [45], prospective studies [20,46], or meta-analyses [23,24,25] have reported an increased atherogenic cardiovascular risk in CD patients, especially related to coronary or peripheral districts. Moreover, many of these studies have emphasized an overall increased frequency of established CVD factors such as hypertension [47], dyslipidemia [48], and diabetes mellitus [49] among CD patients. Mechanistically, these findings are explained by the continuous release of inflammatory mediators from the injured gut, which has a systemic effect in addition to the local one and can damage the vascular endothelium and other structures of the cardiovascular system [50]. Another possible factor increasing the CVD risk in CD patients could be related to GFD, which was suspected to represent a risk factor for atherosclerosis *per se* [51]. The main differences between our findings and the results of these studies, as reported by the authors themselves, could rely on the small sample size, short duration, lack of controls, and missing to adjust for relevant confounders, including sex and age. More importantly, there was no control over the duration of GFD, given the fact that gluten is considered a potential pathogenetic trigger for atherogenesis, through the autoimmune inflammatory processes in CD patients.

While the mainstream scientific literature reports an increased CVD risk among celiac patients, on the contrary, studies supporting a null or reduced risk are not very numerous [16,23,24,27,46,52,53]. In an early prospective multicenter study recruiting 1072 Italian adult celiac patients, Corrao et al. reported a nonsignificant reduced cardiovascular risk despite a two-fold increase in overall mortality [46]. In 2004, West et al. published a large population-based study (in 3590 CD patients) in which a reduction of nearly 15% of myocardial infarction risk compared to controls was observed, although the risk of stroke was increased by 30% [16]. Similarly, Anderson et al. reported significantly lower mortality from circulatory system disease in the anti-gliadin antibody (AGA) positive subgroup of celiac patients [Stardardized mortality ratio (SMR) 0.70, 95% CI 0.49–0.91] but not in the AGA positive/endomysium antibody (EMA)-negative subgroup [SMR 0.77, 95% CI 0.45–1.09] [53]. More recently, an open cohort study detected a slightly lower cumulative incidence of cardiovascular deaths (35 vs. 51 per 10,000 person years) among 10,825 celiac patients from the UK [52]. Finally, a Mendelian randomization study aiming to investigate the causal effect of CD on CVD failed to provide evidence supporting a causal association of CD with a number of atherosclerosis-based disorders, including ischemic stroke and peripheral artery disease [27]. In support, the lack of association between CD and CVD was also described in the meta-analyses of Emilsson et al. [23] and Heikkilä et al. [24]. In accordance with these investigations, the results of our study, conducted in a population with a high frequency of CD, indicate that the disease significantly lowers the risk of CVD even after adjusting for traditional risk factors. Based on what is known about the pathophysiology of CD, some speculations can be advanced to explain our results.

In general, the association of CD with CVD is attributed by most authors to three main factors, namely (i) an excess of traditional cardiovascular risk factors among celiac patients; (ii) the presence of a chronic subclinical inflammation that can fuel systemic vascular damage and stimulate atherogenesis, and (iii) the peculiar psychological attitude of celiac patients with regard to their own health.

The CVD is multi-factorial, and its occurrence is related to the presence of traditional risk factors, including non-modifiable ones (sex, age, ethnicity, family history) and modifiable ones (smoking, diet, type 2 diabetes, dyslipidemia, physical activity), among others [54]. Additional possible risk factors have also been identified [36]. These various risk factors and their variable interactions may potentially modify the relationship between CD and CVD.

An increased risk of high blood pressure was reported by Lim et al. in a CD case report [55]. Hypertension was attributed to hyperhomocysteinemia resulting from increased intestinal permeability, which is supported by the reversibility of hypertension following a B12-enriched GFD. On the other hand, West et al., in 3790 CD adults compared with 17,925 age- and sex-matched controls, observed a reduced prevalence of hypertension (11% vs. 15%), OR 0.68 (95%CI 0.60–0.76) [56]. Similarly, in the present study, the frequency of arterial hypertension was reduced in CD patients compared to controls (15.2% vs. 26.7%, *p* < 0.0001). Moreover, when the analysis was repeated, including hypertension among covariates, the protective effect of CD on CVDs remained.

Celiac patients, including undiagnosed cases, may have a lower attitude toward smoking and thus would be protected from premature atherosclerosis [57]. This feature was evident also in our cohort; however, only the current smoking status, not that of a former smoker, was associated with increased vascular risk; in any case, the protective effect of CD persisted after correction for the smoking habit.

In most populations with available data, CD favors dyslipidemia among other hallmarks of the metabolic syndrome [58]. An altered lipid profile has been attributed to GFD adoption [51], despite the general tendency to minimize the impact of the diet on the global cardiovascular risk of CD patients. In a previous article, we argued that the concern about increased CVD risk in patients following GFD was overemphasized and that untreated celiac patients show rather low or modest total cholesterol levels [59]. Indeed, in the present study, the frequency of dyslipidemia was slightly lower in the CD group than in the controls (9.3% vs. 10.8%). However, in our opinion, this difference, albeit significant, is not sufficient to explain a 70% lower risk of CVD among CD patients.

The existence of a chronic low-level inflammatory state, which can drive atherosclerosis and vascular damage, has been repeatedly implicated as the culprit for increased cardiovascular risk [60]. It has been suggested that patients with untreated CD or those with poor adherence to the GFD, owing to the chronic damage to the intestinal mucosa, elicit an inflammatory reaction with the release of several cytokines into circulation. Moreover, CD patients could experience nutritional unbalance, including folate deficiency, leading to hyperhomocysteinemia [61], a further driver of atheromas [62]. In addition, autoimmune reactions in CD could induce a state of hypercoagulability [63], which may facilitate the formation of thrombi in various vascular districts harboring unstable plaques.

In the present study, these factors were not taken into consideration; however, given the high prevalence of CD in the population, the diagnosis was usually made at an early age and the GFD was promptly initiated. Furthermore, Italian patients with CD—considered a rare condition until a few years ago—are the only ones across the world to benefit from vouchers to obtain gluten-free foods from pharmacies [64]. For this reason, the GFD in Italy is more convenient and easier to adhere to for CD patients.

Actually, our analysis highlighted a weak cardioprotective effect of GFD. On the other hand, a recent Cochrane review provided very little evidence of the role of GFD in the occurrence of CVDs related to gluten intake [65]. Based on the scientific evidence, promotion of GFD among people without CD should not be encouraged.

The third and last mechanism that could explain the lower frequency of vascular involvement in the participants of our study concerns the general attitude of CD patients toward medical self-care. It was observed that celiacs attend visits to their general practitioner more often than members of the general population [66]. Especially in populations with a high prevalence of the disease, such as the one investigated, doctors tend to sensitize their patients to be very attentive to their health in general, besides CD. It can therefore be hypothesized that these patients can manage to avoid potential risk factors for CVD (nutritional and lifestyle) in early adult life. Although in our retrospective study it was not possible to control for these variables, we can at least point out that the frequency with which patients undergo periodic check-ups in our hospital, certainly higher than among non-celiacs, qualifies them as more prone to maintaining optimal health status.

Limitations of this study are its design, which does not allow any causal inference, and the unavailability of socio-economic and nutritional data to refine the statistical models. However, these limitations are partly compensated by the main strength of a well-characterized and well-managed, genetically homogeneous CD cohort. Moreover, it cannot be excluded that the surprisingly lower cardiovascular risk detected in our cohort of CD patients may be due to a lower burden of CVD risk factors in our population. Nonetheless, we feel that these minor differences are too weak to fully explain the findings, and a large part of the effect might be attributed to the particular attention of CD patients toward their own health.

In conclusion, our retrospective study demonstrated that CD reduces the risk of CVD in general and more specifically of carotid lesions after adjusting for potential confounders, especially in those on GFD for a long time. These results deserve future investigations aimed at providing more insight into the association between CD and CVD.

## Figures and Tables

**Table 1:** Descriptive statistics in 8495 study participants according to cardiovascular disease.

Variables	No Cardiovascular Disease (n = 5992)	Cardiovascular Disease (n = 2503)
Sex, n (%)		
Female	3957 (66.0)	1537 (61.4)
Male	2035 (34.0)	966 (38.6) **
Age (years)	46.5 ± 16.1	65.4 ± 11.8
Age, n (%)		
<30	1015 (16.9)	17 (0.7)
30–39	1168 (19.5)	57 (2.3)
40–49	1264 (21.1)	174 (6.9)
50–59	1125 (18.8)	446 (17.8)
60–69	898 (15.0)	817 (32.6)
70–79	442 (7.4)	752 (30.0)
≥80	80 (1.3)	240 (9.6)
Body mass index (kg/m^2^), n (%)		
≥19, <25 (normal weight)	3739 (62.5)	1109 (44.5)
25–29 (overweight)	1686 (28.1)	962 (38.6) **
≥30 (obese)	567 (9.4)	432 (17.0) **
Smoke, n (%)		
No	3441 (57.4)	1290 (51.5)
Former smoker	2035 (33.9)	853 (34.1) **
Current Smoker	516 (8.6)	360 (14.4) **
Dyslipidemia, n (%)		
No	5585 (93.2)	1999 (79.9)
Yes	407 (6.8)	504 (20.1) **
Diabetes, n (%)		
No	5726 (95.6)	2070 (82.7)
Yes	266 (4.4)	433 (17.3) **
Helicobacter pylori, n (%)		
No	4672 (78.0)	1781 (71.2)
Yes	1320 (22.0)	722 (28.8) **
Celiac disease, n (%)		
No	5415 (90.4)	2448 (97.8)
Yes	577 (9.6)	55 (2.2) **

** *p* < 0.01.

**Table 2:** Prevalence of celiac disease according to the different entities of cardiovascular disorders.

Heart Disease	No Celiac Disease	Celiac Disease
Atherosclerosis	563 (22.1%)	8 (13.1%) **
Arrhythmias	141 (5.5%)	3 (4.9%)
Valvulopathy	47 (1.8%)	2 (3.3%)
Cardiomyopathy	5 (0.2%)	1 (1.6%)
Hypertension	1792 (70.3%)	47 (77.0%)

** *p* < 0.01.

**Table 3:** Prevalence of cardiovascular disease in celiac patients according to the duration of a gluten-free diet.

Celiac Disease	No Cardiovascular Disease	Cardiovascular Disease
	(n = 577)	(n = 55)
Gluten–free diet, n (%)		
No	151 (26.2)	23 (41.8) *
<12 months	77 (13.3)	16 (29.1) **
12–23 months	5 (0.9)	1 (1.8)
≥24 months	20 (3.5)	4 (7.3)
Unknown	324 (56.2)	11 (20.0) **

* *p* < 0.05, ** *p* < 0.01.

**Table 4:** Prevalence of CVD risk factors in celiac and non-celiac study participants.

Variables	No Celiac Disease	Celiac Disease
	(n = 7863)	(n = 632)
Body mass index, n (%)		
<25 kg/m^2^	4372 (55.8)	464 (74.2)
25–29 kg/m^2^	2519 (32.2)	117 (18.7) **
≥30 kg/m^2^	940 (12.0)	44 (7.0) **
Smoke, n (%)		
No	4127 (52.5)	384 (60.8)
Former smoker	2915 (37.1)	205 (32.4) **
Current Smoker	821 (10.4)	43 (6.8) **
Dyslipidemia, n (%)		
No	7011 (89.2)	573 (90.7)
Yes	852 (10.8)	59 (9.3) **
Diabetes, n (%)		
No	7217 (91.8)	579 (91.6)
Yes	646 (8.2)	53 (8.4)
Helicobacter pylori, n (%)		
No	5922 (75.3)	531 (84.0)
Yes	1941 (24.7)	101 (16.0) **

** *p* < 0.01.

**Table 5:** Multivariable regression analysis of the cardiovascular risk according to the presence of celiac disease in 8495 study participants.

Variables	Unadjusted	Adjusted
	OR ^#^ (95%CI ^§^)	OR ^#^ (95%CI ^§^)
Sex		
Female	Ref.	Ref.
Male	1.22 (1.11–1.35) **	1.10 (0.98–1.23) **
Age (y)	1.09 (1.08–1.10) **	1.07 (1.05–1.09) **
Body mass index		
<25	Ref.	Ref.
25–29	1.92 (1.73–2.13) **	1.37 (1.21–1.33) *
≥30	2.53 (2.19–2.92) **	1.90 (1.60–2.25) **
Smoke		
No	Ref.	Ref.
Former smoker	1.12 (1.01–1.24) *	1.02 (0.93–1.15)
Current smoker	1.86 (1.60–2.16) **	1.37 (1.19–1.59) **
Dyslipidemia		
No	Ref.	Ref.
Yes	3.46 (3.01–3.98) **	1.80 (1.53–2.12) **
Diabetes		
No	Ref.	Ref.
Yes	4.50 (3.83–5.29) **	2.29 (1.90–2.77) **
Helicobacter pylori		
No	Ref.	Ref.
Yes	1.43 (1.29–1.59) **	1.21 (0.89–1.67)
Celiac disease		
No	Ref.	Ref.
Yes	0.21 (0.16–0.28) **	0.30 (0.22–0.41) **

* *p* < 0.05, ** *p* < 0.01; ^#^ Odd Ratio, ^§^ Confidence Interval.

**Table 6:** Carotid parameters obtained by ultrasonography in 333 patients according to the presence of celiac disease.

Variables	No Celiac Disease	Celiac Disease
	(n = 299)	(n = 34)
Mean carotid cross-sectional area (mm^2^)		
	12.93 ± 3.97	9.95 ± 3.88
Mean Intima-media thickness (mm)		
	0.74 ± 0.14	0.61 ± 0.21 **
Right carotid plaques, n (%)		
No	142 (47.5)	24 (70.6)
Yes	157 (52.5)	10 (29.4) *
Left carotid plaques		
No	149 (49.8)	30 (88.2)
Yes	150 (50.2)	4 (11.8) **
Any carotid plaques		
0	115 (38.5)	26 (76.5)
1	64 (21.4)	4 (11.8) *
2	120 (40.1)	4 (11.8) *

* *p* < 0.05, ** *p* < 0.01.

## Data Availability

The data presented in this study are available on request from the corresponding author.
